# Population Incidence and Burden of Juvenile Idiopathic Arthritis on Australian Health System: Data Linkage Study

**DOI:** 10.1111/jpc.70098

**Published:** 2025-05-31

**Authors:** S. J. Lain, L. Ivancic, J. Chaitow, D. Singh‐Grewal, D. M. Bond, A. Von Huben, R. Colagiuri, S. Colagiuri, N. Nassar

**Affiliations:** ^1^ Child Population and Translational Health Research, Faculty of Medicine and Health The University of Sydney Australia; ^2^ Leeder Centre for Health Policy, Economics and Data, Faculty of Medicine and Health The University of Sydney Australia; ^3^ Department of Rheumatology the Sydney Children's Hospitals Network Sydney New South Wales Australia; ^4^ Department of Rheumatology John Hunter Children's Hospital Newcastle New South Wales Australia; ^5^ Paediatrics and Child Health The University of Sydney Sydney New South Wales Australia; ^6^ Discipline of Paediatrics and Child Health University of NSW Sydney Australia; ^7^ Juvenile Arthritis Foundation Australia (JAFA) Sydney Australia; ^8^ Boden Initiative, Charles Perkins Centre The University of Sydney Australia

**Keywords:** adolescent, general paediatrics, pain

## Abstract

**Aim:**

There is a lack of population‐based information on patterns of healthcare for children with juvenile idiopathic arthritis (JIA). The aim of this study was to examine the population incidence of health service utilisation for children with JIA.

**Methods:**

We conducted a population‐based data linkage study that examined children aged < 16 admitted to hospital with a diagnosis of JIA in New South Wales, Australia between 2002 and 2019. The annual incidence of JIA hospitalisations was calculated using population denominators. Health service utilisation and associated costs to the health system 12 months before and after the first JIA admission were examined using linked hospital admission, emergency department (ED) and outpatient datasets.

**Results:**

A total of 1433 children were admitted to hospital with a first diagnosis of JIA; the highest annual incidence was 7.2/100 000 children. In the year before the first JIA admission, 29% were admitted to hospital, 43% presented to the ED and 48% attended an outpatient clinic. In the year following the first JIA admission, inpatient/outpatient attendance increased: 44% had at least one inpatient admission, 61% attended ≥ 1 outpatient clinics. ED presentations remained stable. The unadjusted total cost to the health system in the year before the first JIA admission was $4422 per child diagnosed with JIA compared to $11 806 in the year following the first JIA admission.

**Conclusion:**

Children with JIA have been demonstrated to be frequent users of hospital services, particularly just before and following their first admission for JIA, highlighting the impact of JIA both on the child and the health system.

**Summary**

- What is already known on this topic
  ○ Juvenile idiopathic arthritis (JIA) is the most common rheumatological disease of childhood.
  ○ The pathway to diagnosis of JIA can be complicated and involve numerous health professionals.
  ○ Children with JIA often have comorbidities and side effects from treatment that greatly impact their quality of life.
- What this study adds
  ○ This paper provides a population‐based estimate of the incidence of JIA hospital admissions in New South Wales.
  ○ Children with JIA are frequent users of health services, but health service utilisation fluctuates across the course of the condition with increased usage around first hospital admission for JIA.

## 1 Background

Juvenile idiopathic arthritis (JIA) is the most common rheumatological disease of childhood. It refers to a group of autoimmune diseases characterised by joint pain, swelling, stiffness and inflammation occurring before the age of 16 years [1]. In Australia, it is estimated that between 1 and 4 in 1000 children live with JIA [2, 3], there are no population‐based data collected on children with JIA. Parents of children with JIA have highlighted the importance of reporting incidence and prevalence rates, as it helps to understand that they are not alone and raise awareness of the condition [4].

There is also a lack of population‐based information on health service utilisation of children with JIA and the patterns of healthcare they access. A scoping review found that surveys were most frequently used to obtain health resource use amongst children with JIA, and most studies examined a time frame of 1 year [5]. Access to health services changes across the course of the disease, including diagnosis, stabilisation of medication and flare ups; hence, information is needed to better identify the burden of JIA on the health system. A Canadian study examined health service utilisation for 389 children with JIA across an 11‐year time frame [6]; however, there have not been studies examining patterns of health service utilisation in Australia.

The primary aim of this study was to examine the population incidence of health service utilisation for children with a diagnosis of JIA. In particular, we examine hospital inpatient, outpatient attendance and emergency department (ED) presentations around the time of first admission with JIA. We also sought to identify characteristics of children who present to hospitals, comorbid conditions and procedures undertaken in the hospital setting.

## 2 Methods

### 2.1 Study Design and Study Population

We conducted a population‐based data linkage study that examined children aged less than 16 years admitted to hospital with a diagnosis of JIA in New South Wales (NSW), Australia between 2002 and 2019. Cases of JIA were identified from the NSW Admitted Patient Data Collection (APDC), which is a census of all hospital admissions in NSW. The APDC includes information on diagnoses coded according to the International Classification of Diseases‐Australian Modification (ICD‐10‐AM) and procedures undertaken, classified using Australian Classification of Health Interventions, Australian Modification (ACHI).

JIA was identified from ICD‐10‐AM diagnosis codes assigned to each hospital admission. Each hospital admission record includes a principal diagnosis and up to 50 additional diagnoses. A diagnosis of JIA is only recorded when a patient is receiving treatment or procedures for JIA, or hospitalised for another condition of which JIA impacts the hospital stay. Two paediatric rheumatologists (J.C. and D.S.) identified six ICD‐10‐AM diagnosis codes relevant to JIA (see Table S1). In addition, children with a diagnosis of ‘other arthritis’ recorded (M13) (without any other record of JIA codes) were examined separately. Although this code should not be used for JIA, it has been included in past definitions of JIA [7].

### 2.2 Data Sources

Once the cohort of JIA children was identified from the APDC, all hospital admissions up to age 16 years were examined. Hospital admissions of identified cases were linked to ED presentations and outpatient clinic visits in NSW. ED visits to public hospitals from 2005 to 2019 are captured in the NSW Emergency Department Data Collection (EDDC). The principal diagnosis of each ED presentation is recorded using ICD9, ICD10‐AM and SNOMED codes. These codes were grouped into broad diagnostic groups (see Tables S1 and S2). The non‐admitted patient (NAP) data collection captures visits to outpatient clinics at NSW hospitals from 2016 to 2019.

### 2.3 Study Factors

Demographic characteristics were identified from the hospital admission record. These included: age at first admission with a diagnosis of JIA, sex, country of birth, health insurance status and postcode. Geographical remoteness and socioeconomic status (SES) were obtained using residential postcode. Remoteness of residential area was derived using the Australian Statistical Geography Standard classification of Accessibility/Remoteness Index of Australia [8], and SES was derived using the Australian Bureau of Statistics Index of Relative Socio‐Economic Advantage Disadvantage [9].

### 2.4 Data Analysis

The number of children admitted to hospital each year for the first time with a JIA diagnosis was identified for the years 2005–2019. The years before 2005 were used as a look‐back period to ensure the first JIA admission was identified. The annual incidence of hospitalisations (per 100 000) for children with a new admission for JIA was calculated, with annual population figures for NSW used as the denominator in incidence calculations [10]. The total number of hospital admissions and ED presentations each year was identified. The annual incidence of children with only a diagnosis of ‘other arthritis’ was assessed separately.

We next examined the demographic characteristics, the presence of comorbidities and health service use for the complete cohort of those diagnosed with JIA (2002–2019), excluding children with ‘other arthritis’. The characteristics of those with a first admission for JIA and those for all hospital admissions were compared. Comorbid conditions (based on primary diagnoses excluding JIA), procedures undertaken during hospital admissions, total ED presentations and visits to outpatient clinics were all identified. To examine the timing of health service utilisation relative to the first JIA admission, a cohort of children with their first JIA admission from 2008 to 2015 were identified. For these children, hospital admissions and ED presentations in the 3 years before, and 3 years post the first admission for JIA were calculated.

To examine costs of health service use before and after first JIA admission, the total and average number of hospital admissions, ED presentations and outpatient visits in the 12 months before and 12 months following the first JIA admission were identified. As outpatient data was only available for 4 years (2016–2019), to examine use of outpatient clinics relative to first JIA admission, those who had their first JIA admission recorded in 2017 were identified. The cost of inpatient, outpatient, and ED presentations from the perspective of the Australian government were based on published costs in the National Hospital Cost Data Collection Public Sector Report (2018–19) [11] and inflated to 2023/2024 prices based on the increase in National Efficient Price, the cost benchmark for hospital funding in Australia [12]. All inpatient admissions are classified into an Australian Refined Diagnoses Related Group (AR‐DRG) based on diagnoses information. The average cost per AR‐DRG was used for inpatient admissions, which is influenced by many factors, including the complexity of the admission and demographic factors of the patients treated. Total cost of all health service attendances was aggregated and average cost per child with a diagnosis of JIA calculated.

All analyses were completed in SAS, version 9.4 software (SAS Institute).

## 3 Results

**FIGURE 1:**
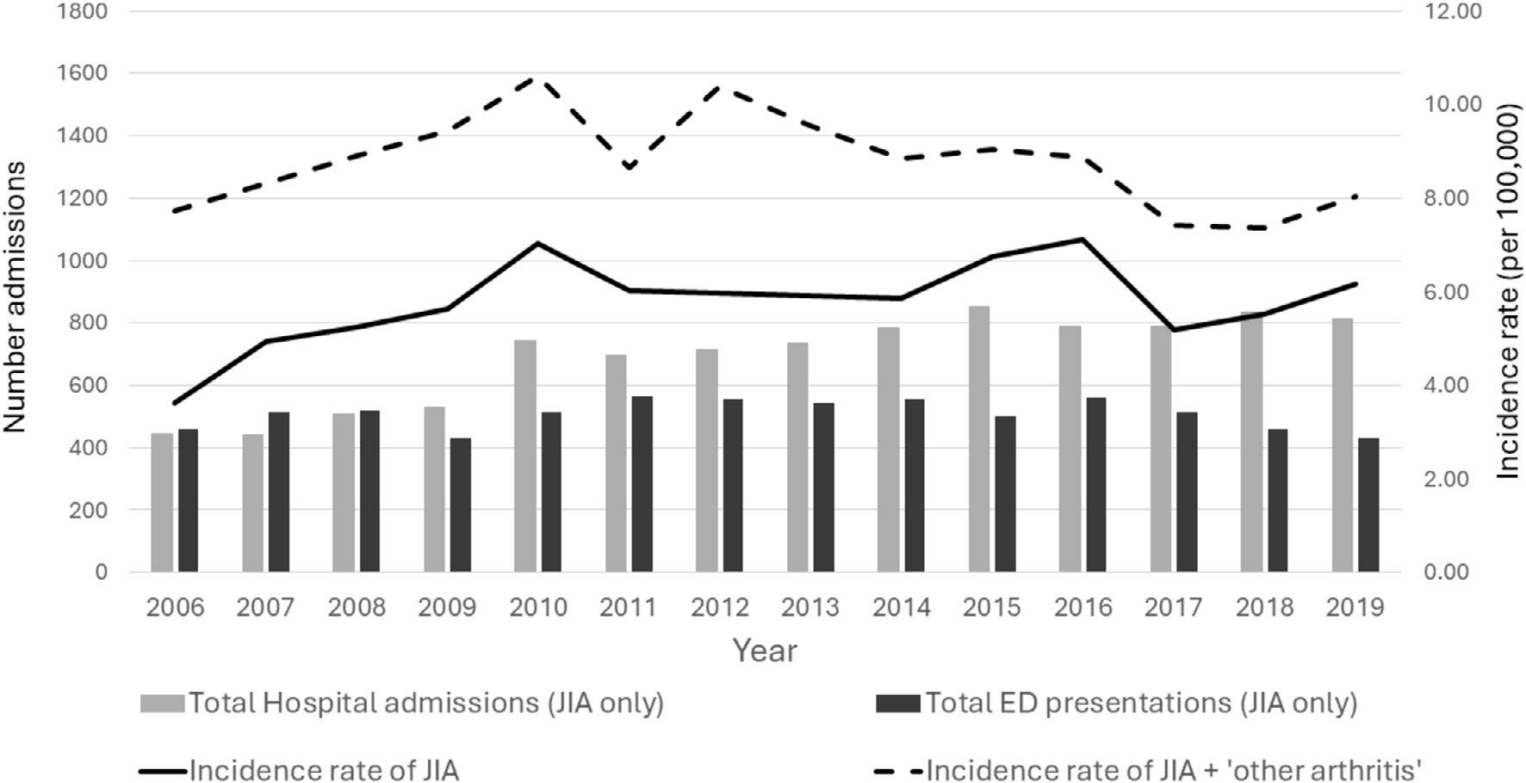
Annual incidence of JIA admissions, total hospital admissions and emergency department presentations in NSW hospital, 2006–2019.

Between 2002 and 2019 there were 1433 children admitted to hospital in NSW with a recorded diagnosis of JIA, 92.1% had a diagnosis of M08 (juvenile arthritis) recorded. In addition, there were 834 children admitted to hospital with only a diagnosis of ‘other arthritis’ (M13) recorded. There was an increase in the number of children admitted to hospital with a first JIA diagnosis over time from around 50 to 110 per year, giving an overall incidence rate for JIA of 5.4 of 100 000 children, with a peak of 7.2 of 100 000 in 2016 (Figure 1). When a recorded diagnosis of ‘other arthritis’ (M13) was also included, the overall incidence was 8.5 of 100 000 children per year, with a peak of 10.6 of 100 000 children in 2010 (Figure 1). The proportion of children with a diagnosis of ‘other arthritis’ recorded decreased over the study period.

The 1433 children in the cohort that had a recorded admission diagnosis of JIA had a total of 11 581 hospital admissions during the study period (Table 1). The cohort was 59.9% female, 43.3% had their first JIA admission aged 5–11 years (median age of 8 years), and 42.2% of first JIA admissions involved a presentation to the ED (Table 1). Comparatively, a higher number of those with only ‘other arthritis’ recorded were male (58.4%), resided in an area of lower SES and had more ED involvement (80.5%) (Table 1).

**TABLE 1:** Demographic characteristics of children with juvenile idiopathic arthritis and other arthritis, and distribution of characteristics across all hospital admissions for JIA cohort 2002–2019.

Child demographic characteristics	Children with diagnosis of JIA (*N* = 1433)	Total admissions for JIA cohort (*N* = 11 581)	Children with Other arthritis only (*N* = 834)
	*n* (%)	*n* (%)	*n* (%)
Age at first JIA admission			
0–4 years	449 (31.3)	2493 (21.5)	319 (38.3)
5–11 years	621 (43.4)	5749 (49.7)	337 (40.4)
12–15 years	363 (25.3)	3339 (28.8)	178 (21.3)
Sex			
Female	858 (59.9)	6765 (58.4)	347 (41.6)
Male	575 (40.1)	4816 (41.6)	487 (58.4)
Country of birth			
Australia	1332 (93.0)	11 127 (96.1)	783 (93.9)
Other	100 (7.0)	446 (3.9)	51 (6.1)
Socioeconomic status			
Most disadvantaged (< 20th quintile)	255 (17.8)	2343 (20.2)	208 (24.9)
20th–39th quintile	206 (14.4)	1636 (14.1)	139 (16.7)
40th–59th quintile	251 (17.5)	1850 (16.0)	155 (18.6)
60th–79th quintile	276 (19.3)	2841 (24.5)	134 (16.1)
Most advantaged (≥ 80th quintile)	396 (27.6)	2625 (22.7)	172 (20.3)
Missing	49 (3.4)	286 (2.5%)	26 (3.1)
Remoteness			
Major city	1007 (70.3)	8683 (75.0)	545 (65.3)
Inner regional	257 (17.9)	1627 (14.0)	175 (21.0)
Outer regional, remote	121 (8.4)	994 (8.6)	88 (10.6)
Missing	48 (3.3)	277 (2.4)	26 (3.1)
Payment status (health insurance)			
Public	727 (50.7)	6872 (59.3)	593 (71.4)
Private	687 (47.9)	4597 (39.7)	232 (27.9)
Other/unknown	19 (1.4)	112 (0.9)	9 (1.1)
Emergency department involvement			
Yes	460 (32.1)	2524 (21.8)	648 (80.5)
No	630 (44.0)	7796 (67.3)	157 (19.5)
Unknown	343 (23.9)	1261 (10.9)	29 (3.5)

For the cohort of JIA (excluding ‘other arthritis’), when admissions with a primary diagnosis of JIA (53% of admissions) were excluded, the most common comorbid conditions identified were diseases of the musculoskeletal system and connective tissue (14.6%), diseases of the digestive system (13.8%) and diseases of the eye and adnexa (11.4%) (Table 2). The most common procedure performed was the administration of pharmacotherapy (34.3%). From 2016 to 2019 there were almost 30 000 occasions of service at hospital outpatient clinics (Table 2).

**TABLE 2:** Top five diagnoses and procedures for hospital admission, diagnoses for ED presentation, outpatient clinics for those with a JIA diagnosis (*n* = 1433).

	*n* (%)
All admissions	
Principal diagnosis ICD10 chapters (top diagnosis—excluding JIA diagnosis)	*N* = 5497
M chapter: Musculoskeletal (M25—pain or swelling in joint 18%)	803 (14.61)
K chapter: Digestive system (K50—Crohn disease: 50%)	760 (13.83)
H chapter: Diseases of eye (H20—iridocyclitis: 55%)	626 (11.39)
J chapter: Respiratory system (J45—asthma: 17%)	544 (9.90)
R chapter: Symptoms (R50—fever 24%)	449 (8.17)
Principal procedure	*N* = 11 581
Administration of pharmacotherapy	3975 (34.3)
Administration of agent into other musculoskeletal sites	1331 (11.5)
Aspiration of other musculoskeletal sites	657 (5.7)
Injection of infusion of therapeutic or prophylactic substance	591 (5.1)
Generalised allied health interventions	341 (2.9)
Emergency department presentations	*N* = 6910
Infection	1560 (22.6)
Accident	1367 (19.8)
Joint pain, arthritis	850 (12.3)
Digestive system	591 (8.6)
Symptoms (including fever, headache, rash, etc.)	529 (7.7)
Outpatient clinics (2016–2019)	*N* = 29 899
Rheumatology	5064 (16.94)
Paediatrics/paediatric medicine	4496 (15.04)
Gastroenterology/enteral nutrition	2670 (8.93)
Ophthalmology/orthoptics	2573 (8.61)
Allied health^a^	2261 (7.56)

^a^ Allied health clinic included: physiotherapy, occupational therapy, speech pathology, podiatry.

**FIGURE 2:**
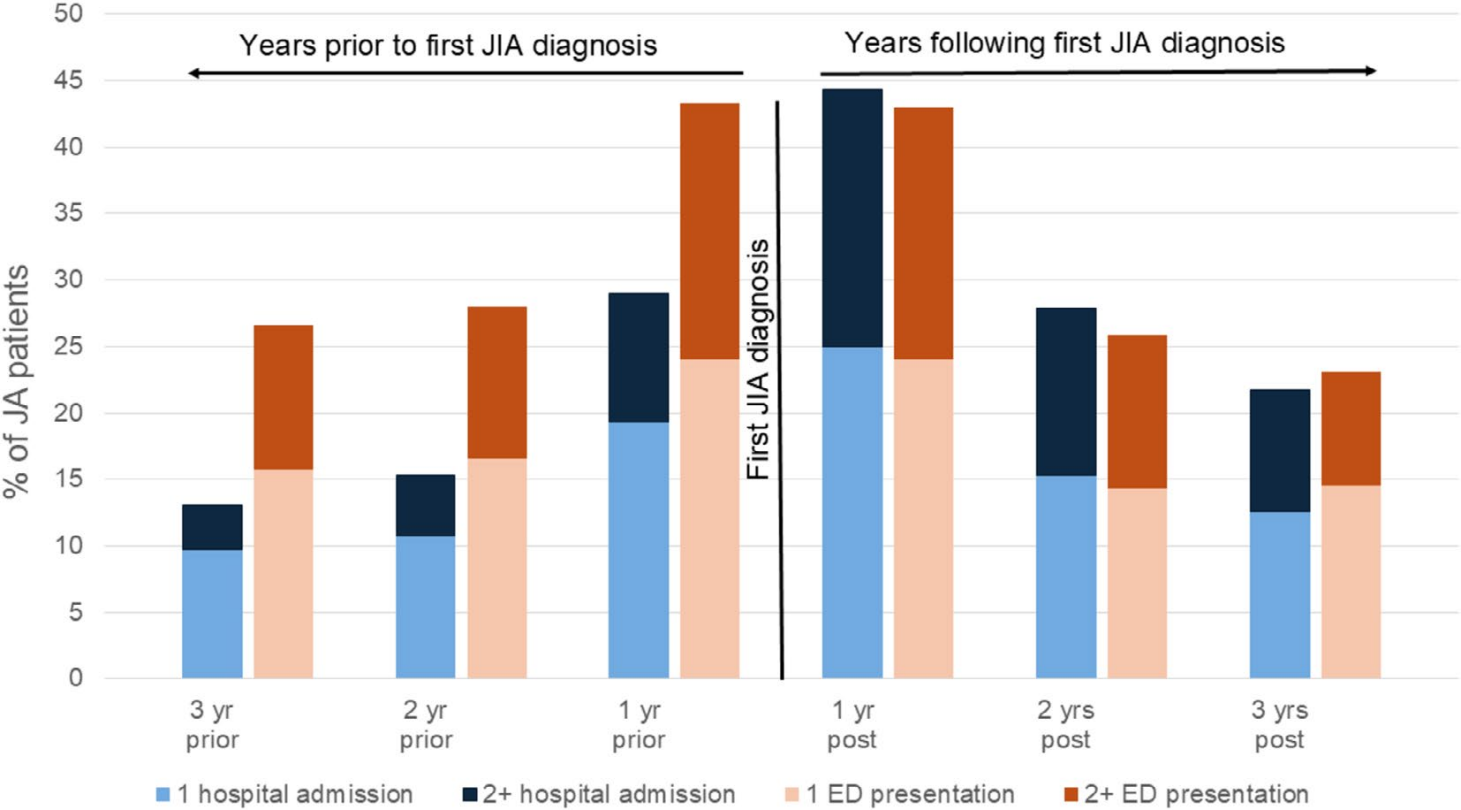
Proportion of JIA patients with ED and hospital admissions per year, in the 3 years before and following the first JIA admission in NSW for those diagnosed with JIA 2008–2016.

There were 673 children with a first JIA admission between 2008 and 2015. In the year before the first JIA admission, 43% presented to the ED, with almost one fifth (19%) of all children presenting to the ED more than once. In the year following the first admission with a JIA diagnosis, inpatient admissions were highest, with 44% of the cohort having at least one hospital admission in addition to the initial JIA inpatient admission (Figure 2).

In the year before the first JIA admission, 48% of children attended an outpatient clinic and 61% attended an outpatient clinic following their first JIA admission, having an average of 10 visits (Table 3). The unadjusted total cost to the health system per child with a diagnosis of JIA in the year before the first JIA admission was (inpatient $2696 + ED $847 + outpatient $879; Table 3) $4422 compared to (inpatient $8754 + ED $815 + outpatient $2237; Table 3) $11 806 in the year following first JIA admission, including the inpatient admission with the first JIA diagnosis.

**TABLE 3:** Burden and unadjusted cost to the health system of the JIA cohort the year before and year after first JIA admission.

	Hospital inpatient admissions		Emergency department presentations		Outpatient attendances	
	12 months before first JIA admission	First JIA admission and readmissions in post 12 months	12 months before first JIA admission	12 months post first JIA admission	12 months before first JIA admission	12 months post first JIA admission
Average visits per person (SD)	0.52 (1.19)	2.18 (3.46)	1.01 (1.51)	0.88 (1.38)	3.83 (8.10)	10.45 (16.50)
Mean cost per child (SD) per year	$2696 ($6284)	$8754 ($14 776)	$847 ($1073)	$815 ($1071)	$879 ($1443)	$2237 ($2796)
Top three principal diagnosis/outpatient clinic	Joint disorder, M25 (12.3%)	Juvenile arthritis, M08 (70%)	Joint pain/arthritis (26.1%)	Joint pain/arthritis (22.5%)	Paediatric medicine (24.9%)	Rheumatology (33.2%)
	Other arthritis, M13 (8.6%)	Crohn's disease, K50 (3.1%)	Infection (19%)	Accident (18%)	Rheumatology (19.8%)	Paediatric medicine (21.0%)
	Crohn's disease, K50 (5%)	Other rheumatoid arthritis, M06 (1.8%)	Accident (17%)	Infection (15%)	Orthopaedics (7.8%)	Allied health (10.7%)

## 4 Discussion

This study examines the annual incidence of health service utilisation of a cohort of children admitted to hospital with a first diagnosis of JIA. To our knowledge, this is one of the largest cohorts examined to explore the burden of JIA on the Australian health system and shows that contact with the health system is multifaceted and fluctuates, potentially in response to the course of the condition or its treatment. Health service utilisation is greatest in the year following the first JIA hospital admission and is reflected by corresponding healthcare costs.

The highest annual incidence rate of first admission to hospital with JIA reported in this study was 7.2 of 100 000, and 10.6 of 100 000 children when ‘other arthritis’ was included. Studies using inpatient data in Western Australia (7.9/100 000) [13] and the United States (7.4/100 000) [14] report similar incidence admission estimates; however, hospital inpatient data underestimate incidence rates of JIA diagnosis compared to other forms of case ascertainment [15]. Data collected from clinical practice records reported incidence rates from 11 to 17 per 100 000 in the United Kingdom [16], Quebec [17] and Nordic countries [18]. An audit of private and public paediatric rheumatology specialist clinical records or a nationally representative register would better identify the incidence of JIA in Australia.

Patterns of healthcare utilisation change over the course of the disease. In the year before the first JIA admission, almost half of the cohort presented to the ED. A Canadian study found that 12 months before diagnosis of JIA, attendance at ED and general practitioners (GP) peaked as families sought help before referral to specialists [6]. The path to diagnosis may be long and complicated [19]. In Australia, the average time to diagnosis from symptom onset is 11 months, with an average of 2.7 health professionals seen [20]. Directly following diagnosis, health service utilisation is high as newly diagnosed children begin treatment and try to stabilise symptoms [17]. The first year following diagnosis requires intensive medical management, with children cycling through multiple medications and many having flares [21]. Early aggressive treatment has been shown to dramatically improve outcomes [22] and could reduce inpatient hospital admissions. In NSW, this management of children with JIA occurs in an environment that has a considerable shortfall of paediatric rheumatologists [23], which is a major barrier to providing timely and accessible care for the children [24].

The burden of JIA on the health system extends beyond the management and treatment of joint pain and swelling, with comorbidities related to JIA or symptoms associated with treatment that impact health service utilisation. One of the most common comorbidities is uveitis, with reported prevalence of 15%–20% of young people with JIA [25]. Issues with the digestive system are also increased amongst children with JIA [26] and gastrointestinal side effects of treatment with methotrexate are common [20]. Although arthritis is a common comorbidity for children with inflammatory bowel disease, such as Crohn's disease [27], 92% of our cohort had the ICD‐10‐AM code for primary JIA recorded. These diverse health issues that children with JIA have require a range of health services. The standards of care for the management of JIA recommend a multidisciplinary team including paediatric rheumatologists, physiotherapists, occupational therapists and ophthalmologists [28]. This multidisciplinary care can be accessed at some public hospital outpatient clinics; however, to observe the complete picture of healthcare accessed by children with JIA, including in private practice settings, additional datasets are required.

A recent systematic review assessing the economic impact of JIA found the majority of direct healthcare costs related to JIA were derived from medication and medical appointments, and highlighted the importance of examining costs at different time points during the course of JIA [29]. Our study found that the direct hospital‐related healthcare costs in the year following the first hospital admission with a JIA diagnosis were over 2.5 times the healthcare costs for the year prior, similar to a study performed in the Canadian health system [6]. Healthcare costs for children with JIA in our study were from the perspective of the Australian government and only included hospital‐related costs. An Australian survey of children with JIA and their families in 2023 found annual in‐hospital costs were $12 771, similar to the total hospital costs the year following the first JIA admission [20]. When medications, diagnostic testing, and specialist visits are included, costs to the Australian government are estimated at $24 396 per person per year [20]. Out‐of‐pocket costs for families have been estimated at an additional $4179 [20] highlighting that JIA has a huge impact on both the healthcare system and families.

The main strength of this study is a large, population‐based cohort that spans 18 years. The data linkage of numerous datasets allows the examination across different health services, and the data linkage within datasets ensures health service usage per person is captured, rather than only capturing total hospital admissions [7]. The main limitation of this study is the identification of children with JIA, via a recorded diagnosis in a hospital admission, which will miss cases of JIA managed outside the hospital. We also do not have data about private specialists and GPs attendance and medication data to get a complete picture of the burden on the health system; however, this study does identify the burden of JIA on the public hospital system in NSW.

In conclusion, this study presents contemporary incidence rates of JIA hospital admissions and the total burden of JIA on hospitals in NSW. Children with JIA are frequent users of health services, particularly in the year before and just following their first hospital admission with JIA. This highlights the impact of JIA on the child and the health system and provides data to assist with planning of health services for this group of children.

## Ethics Statement

Ethics approval for the study was attained from the NSW Population and Health Services Research Ethics Committee (2019/ETH11532).

## Conflicts of Interest

The authors declare no conflicts of interest.

## Supporting information


**Table S1.** ICD10‐AM codes used to identify juvenile idiopathic arthritis.
**Table S2.** Emergency department coding categories.

## Data Availability

The data that support the findings of this study are available from the NSW Ministry of Health, but restrictions apply to data availability, which were used under licence for the current study and therefore are not publicly available.
