# Supplementary material for: Population Incidence and Burden of Juvenile Idiopathic Arthritis on Australian Health System: Data Linkage Study
Source: J Paediatr Child Health. 2025 May 31;61(8):1241–8. doi: 10.1111/jpc.70098 (PMC12397838; doi:10.1111/jpc.70098)
Supplement: Supplementary file 1 — Table S1. ICD10‐AM codes used to identify juvenile idiopathic arthritis. Table S2. Emergency department coding categories. [file JPC-61-1241-s001.docx]

Supplementary Table 1: ICD10-AM codes used to identify juvenile idiopathic arthritis

| ICD10-AM code | Diagnosis |
| --- | --- |
| M05 | Seropositive rheumatoid arthritis |
| M06 | Other rheumatoid arthritis |
| M07 | Psoriatic and enteropathic arthropathies |
| M08 | Juvenile arthritis |
| M09 | Juvenile arthritis in diseases classified elsewhere |
| M45 | Ankylosing spondylitis |

Supplementary Table 2: Emergency department coding categories

| ED Category | Main conditions included | ICD9 codes | ICD10 codes | Top SNOMED codes |
| --- | --- | --- | --- | --- |
| Accident | Injury  Vehicle accident  Bone fracture | 800-999 | Chapters S, T, V, W | 82271004  282026002  44465007  17222009  274164006  312608009  125601008  70704007  111640008  52011008  125596004 |
| Infection | Viral infection  Respiratory infection | 001-139  460-488 | Chapters A, B  J0-J47 | 34014006  281794004  71186008  68566005  17741008  90176007  54150009  111843007  50417007 |
| Arthritis | Arthritis  Joint pain  Swelling of joints  Effusion of joint | 711-719 | M0-M25, M79, S53, R26 | 30989003  239796000  49218002  3723001  202381003  57676002  95854004  247373008  47933007  74323005  299322007 |
| Digestive system | Crohn’s disease  Abdominal pain  Nausea and vomiting | 555-558  787 | K50-K52  R10, R11  A09 | 21522001  422400008  25374005  314212008  14760008  69776003  34000006 |
| Symptoms | Fever  Rash  Headache  Dizziness  Other pain | 780-799 | Chapter R (excluding listed above) | 386661006  25064002  7520000  29857009  63901009  162397003  409089005  404640003 |
